# Placebo Responses and Placebo Effects in Functional Gastrointestinal Disorders

**DOI:** 10.3389/fpsyt.2020.00797

**Published:** 2020-08-25

**Authors:** Paul Enck, Sibylle Klosterhalfen

**Affiliations:** Department of Internal Medicine VI: Psychosomatic Medicine and Psychotherapy, University Hospital Tübingen, Tübingen, Germany

**Keywords:** irritable bowel syndrome, clinical trial, functional dyspepsia, placebo, nocebo

## Abstract

Much has been written about the placebo effects in functional gastrointestinal disorders (FGD), especially in irritable bowel syndrome (IBS), driven by the early hypothesis that in randomized controlled trials (RCTs) of IBS, the placebo effect might be specifically high and thus, corrupts the efficacy of novel drugs developed for this condition. This narrative review is based on a specific search method, a database (www.jips.online) developed since 2004 containing more than 4,500 papers (data papers, meta-analyses, systematic reviews, reviews) pertinent to the topic placebo effects/placebo response. Three central questions—deducted from the body of current literature—are addressed to explore the evidence behind this hypothesis: What is the size placebo effect in FGD, especially in IBS, and is it different from the placebo effect seen in other gastrointestinal disorders? Is the placebo effect in FGD different from other functional, non-intestinal disorders, *e.g.* in other pain syndromes? Is the placebo effect in FGD related to placebo effects seen in psychiatry, *e.g.* in depression, anxiety disorders, and alike? Following this discussion, a fourth question is raised as the result of the three: What are the consequences of this for future drug trials in FGD? In summary it is concluded that, contrary to common belief and discussion, the placebo effect seen in RCT in FGD is not specifically high and extraordinary as compared to other comparable (*i.e.* functional) disorders. It shares less than expected commonalities with the placebo effect in psychiatry, and very few predictors have yet been identified that determine its effect size, especially some that are driven by design features of the studies. Current practice of RCT in IBS seems to limit and control the placebo effect quite well, and future trial practice, *e.g.* head-to-head trial, still offers options to maintain this control, even in the absence of placebos used.

## Introduction

Much has been written—by us (1–4) and by others (5–7)—about the placebo effects in functional gastrointestinal disorders (FGD), especially in irritable bowel syndrome (IBS), driven by the early hypothesis that in randomized controlled trials (RCTs) of IBS, the placebo effect might be specifically high (8) and thus, corrupts the efficacy of novel drugs developed for this condition (9). This has been a popular statement over the next two decades and is still around among many gastroenterologists when explaining the difficulties of IBS RCT and the lack of effective treatment. Previous reviews have attempted to contradict this common belief, but until very recently, a comparison between placebo response rates in IBS, in other functional bowel disorders, in non-functional gastrointestinal disorders and in associated disorders in psychiatry was lacking. While systematic reviews and meta-analyses to estimate the effect size of placebos in comparison to those of drugs were published, a direct comparison of the determinants of the placebo response, *e.g.* in psychiatry, was and is not available.

## Methods

The specific approach taken to assess the relevant papers for the topic of this review is described in more detail elsewhere (10). In an attempt to comprehensively screen the entire medical literature published for papers reporting the placebo effect/placebo response, a PubMed search using the single search term “placebo” was conducted in early 2004. This resulted in more than 100,000 papers at that time. The title and abstracts of these papers were screened retrospectively (at a frequency of maximally 1,000 per day, 7 days a week for about one year), to identify the approximately 1% of all papers relevant for placebo research. These papers were stored in an *Endnote*-like database, respective PDFs were collected, and made available to the local working group in Tübingen. A few years later, a similar search using the term “nocebo” was added. Papers found occasionally and incidentally in book chapters and papers not available *via* PUBMED were added manually to the database, as were papers suggested by colleagues and other researchers.

At the same time (2003), a *prospective* PUBMED search was started that resulted in weekly reports of newly published papers with either of the two terms (on average 200 per week altogether) and again screened for relevance for placebo research. The 1% outcome has increased to about 2% over the years. Overall, this resulted in a database of approximately 4,500 papers (data papers, meta-analyses, systematic reviews, reviews, commentaries, and a limited number of letters) as of mid 2020. These references and monthly updates thereof were made available to the scientific community *via* a newsletter that can be subscribed at <www.jips.online> and has currently a few hundred subscribers. For the purpose of this review and other papers published in the last few years by us and others, this database is screen for new papers relevant to specific topics, such as placebo effects in functional bowel disorders.

So, why another review of the topic, especially in times when drug testing tends to move away from placebo-controlled trials and towards “real life” studies, studies that mimic daily medical routine rather than promote (self-)selection of patients willing to take part in a placebo-controlled test, while others, and presumably the more severely affected patients, prefer open-label treatment, even with novel compounds. Such “observational studies” are experiencing rediscovery and support not only by patients and patient organizations but also by approval authorities. However, eliminating placebos in drug testing does not eliminate the placebo response that is inherent to all medical (and psychological) interventions, even when provided by computerized algorithms—the digital placebo response (11). It has recently been proposed that even with open-label observational studies, proper control of some of the mediators of the placebo response is feasible (12) and thereby insists on a scientific rather than a pragmatic approach.

## Results

In the following, an answer to three major questions that are posed by the continuing discussion is attempted:

A: How large is placebo effect in FGD, especially in IBS, and is it different from the placebo effect seen in other gastrointestinal disorders, such as in IBD?
B: Is the placebo effect in FGD different from other functional, non-intestinal disorders, *e.g.* in other pain syndromes?
C: Is the placebo effect in FGD related to placebo effects seen in psychiatry, *e.g.* in depression, anxiety disorders, and alike?

Following this discussion, a fourth question is raised as a consequence of the three:

D: What are the consequences of this for future drug trials in FGD?

While most of the current knowledge about the placebo effect and the placebo response can be easily accessed *via* the web-platform that was established (www.jips.online) and that currently (end of 2019) contains nearly 4,500 papers (data paper, reviews, meta-analyses) genuinely discussing the placebo effects in medicine (10), final answers are far from being readily available. It may just be that this is *our* final contribution to the discussion.

In the following, the terms placebo effect and placebo response are used more or less interchangeably, but this is in light of the fact that this is a deviation from common practice and definitions [*e.g.* (13)]; for the purpose of this paper it may, however, be acceptable to simplify explanations and ease understanding.

### A: Is The Placebo Effect in FGD (IBS) Different From Other Gastrointestinal Disorders?

The first step to answer this question is to check how large the placebo effect in IBS is, overall and not only in a few but in all studies. According to some meta-analyses the overall size of the placebo effect in IBS is in the range of 40%, be it in conventional drug trials (14), in complementary and alternative medicine interventions (7), or in nutritional interventions (15), with the latter challenged by larger difficulties to maintain some of the standards of good scientific practice, *e.g.* appropriate double-blinding, compliance control, and other features (16).

While attributing 40% of improvement to placebo effects in RCT in IBS seems a lot, this has much to do with the chosen primary endpoints of these studies: Clinical experts and/or approval authorities may have agreed on meaningful degrees of improvement (*e.g.* at least 30% change in average pain rating for one week on a visual analog scale (VAS) between 0 = no pain and 10 = highest imagined—or experienced—pain); the subsequent division of patients into responders meeting these criteria and non-responders simplifies decision making for the benefit of the approval process, but not for clinical routine: are the patients responding with a 29% improvement *only* really non-responders in comparison to the ones with a 30% improvement, and is the patient with the 90% improvement really the same type of responder than the one just meeting the 30% threshold? Dichotomizations of this kind may ignore potentially clinically meaningful differences by reducing data variance, but they ease power calculations, efficacy statistics, and publishing attempts, as well as marketing strategies of the drug. However, for the meta-analyses that have found the 40% to be the average size of the placebo effect, the dichotomization effect may be less pronounced, as long as the same entry criteria into the studies were used. Whether or not this was the case is not as much a consequence of the patient definitions at times (Rom to Rome IV) (17) but rather of the recruitment strategies at the level of the single centers.

**Figure 1:**
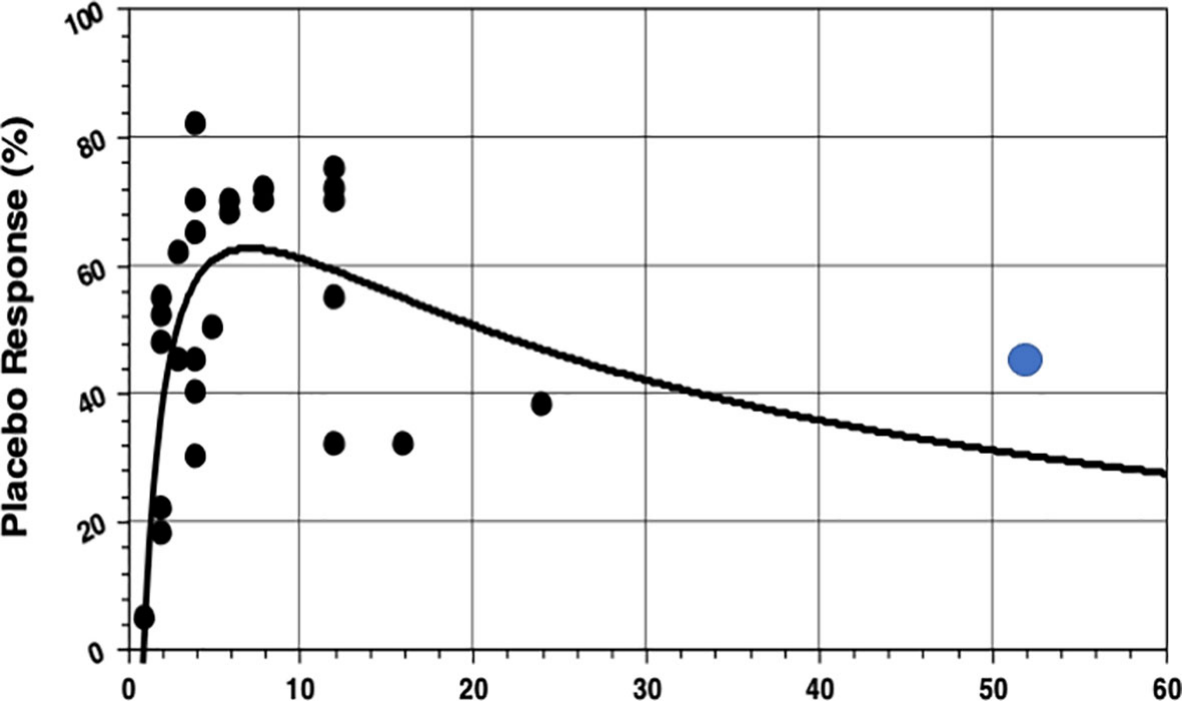
Association between placebo response rates and the duration of treatment in 26 IBS studies from a review (9), supplemented by the first 1-year study (18) (blue dot).The non-linear (rational) regression function is highly significant, but note there are only two studies that lasted longer than 12 weeks at that time. Evidently (what we know now) with longer treatment duration the placebo response rate will be substantially higher (40%) than the 25% in the initial prediction. (Reproduced with permission from Elsevier).

**Figure 2:**
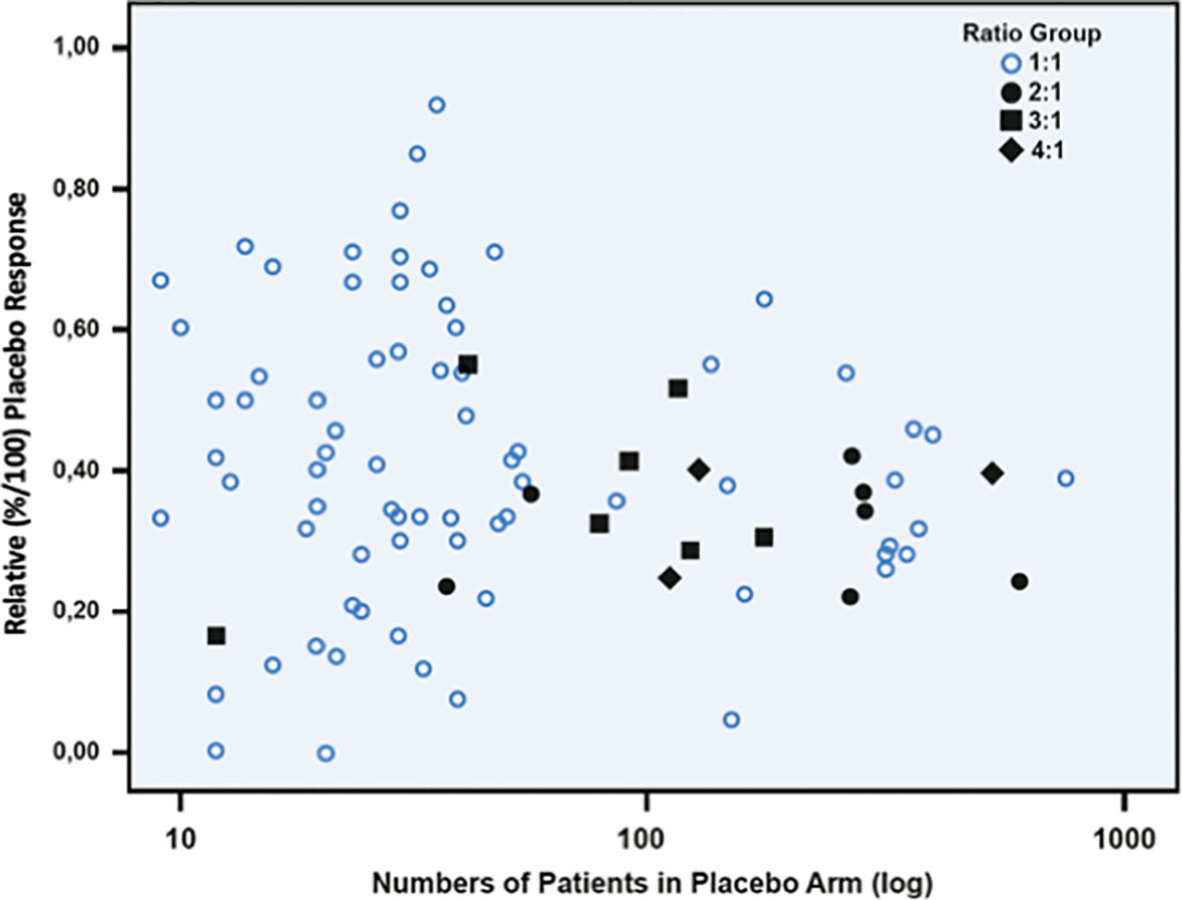
Scatterplot between relative placebo response rates (n/N) and number of patients (log transformed) in the placebo arm of 102 randomized, double-blinded placebo-controlled irritable bowel syndrome studies. It is evident that with sample sizes of more than 100 patients the placebo response tends toward 40%. Open circles indicate studies powered 1:1, and dark marks indicate studies with different unbalanced randomization ratios. (Reproduced from Weimer & Enck (19), with permission from Springer).

**Figure 3:**
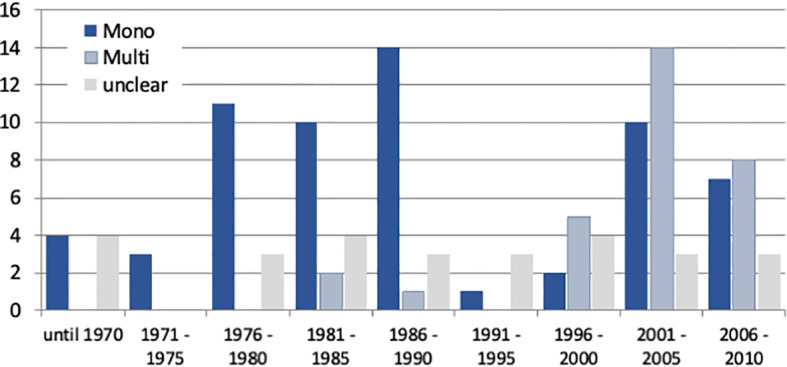
Number of IBS studies published between 1975 and 2010 (data according to Klein (8), Spiller (9), and own data compilations) according to their mono-centric or multicentric nature. Note that monocentric studies dominated until 1990, while multi-center trials became more prevalent thereafter and were the rule after 2010.

The hypothesis that IBS studies yield a higher-than-usual placebo response stems from the times before the Rome definitions of IBS and was first mentioned in a review by Klein (8) as early as 1988. It was Spillers (9) 1999 prediction that the placebo effect would decline to an average rate of 20% once longer studies than the usual 4-week trials were conducted (**Figure 1**). However, as was shown (19), the high placebo response rates in earlier studies were not as much a function of the trial duration but rather a function of the number of patients included (**Figure 2**). Small sample sizes carried the risk of higher variability of the placebo effect across studies, and it was the trials with the highest response rates that drove the impression and stuck in peoples mind; on average, the placebo response was always around 40%. Another fact that may have driven higher placebo response rates in individual RCTs was the fact that most of these studies were single center trials, while multi-centric studies became only the rule after 2000 (**Figure 3**). In monocentric studies, a single empathic doctor can eliminate the entire drug effect by raising the placebo response, especially with small samples, while nowadays block-randomization prevents or at least minimizes a disbalance in efficacy between centers.

**Figure 4:**
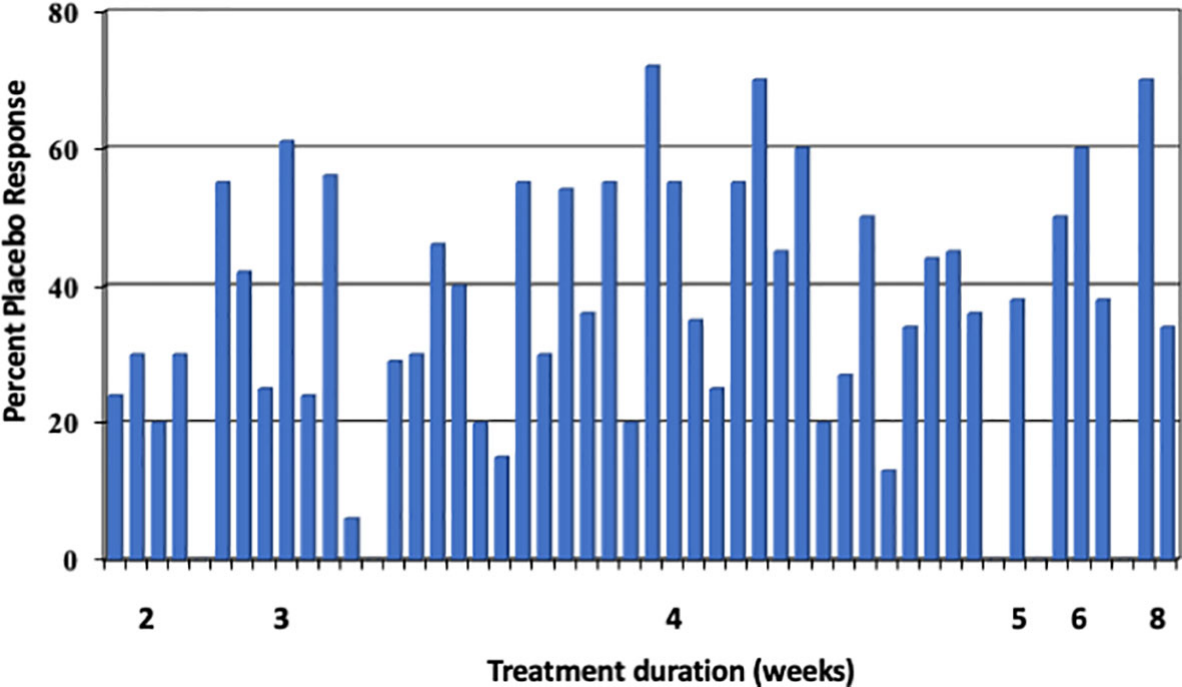
Placebo response rates (in %) in 29 functional dyspepsia studies [data according to Mearin et al. (21) and Allescher et al. (20)], sorted according to length of study (in weeks). Each bar represents one study. The mean placebo response across all 45 trials is 40%. (Reproduced from Enck & Klosterhalfen (1), with permission from Wiley).

Much less is published about the placebo effects in RCT with FGD other than IBS, *e.g.* in functional dyspepsia (FD), but a systematic review from 2001 (20) yielded an overall placebo response rate of 230/619 (37.2%) patients with functional (“non-ulcer”) dyspepsia in 19 studies with gastroprokinetics, and it was 350/754 (46.4%) in 10 studies with acid blocking agents, resulting in an overall placebo response of 42.2%. The placebo effect varied between 6 and 73% (**Figure 4**) and therefore, was quite similar to the IBS studies at the time (3 to 83%) (22). This was noted by others as well (23) but has not (yet) led to an updated meta-analysis of the response rate across all (or many) trials. A 2018 systematic review and meta-analysis of 43 prokinetic RCT in functional dyspepsia (24) noted a 60% risk to be not symptom-free after prokinetic treatment compared to a 74% risk after placebo, with a rather high risk of bias in many studies. Thus, in FD the placebo response seems to be of similar size to that in IBS RCT. However, predictor analyses of the placebo response in FGD other than IBS have never been performed.

A 2015 systematic review of placebo response rates across many medical conditions (25) listed other gastrointestinal diseases, such as gastric and duodenal ulcers, reflux disease, and inflammatory bowel diseases that were meta-analyzed—a few additional meta-analyses have been published ever since (see **Table 1**). As can be seen, compared to IBS, the placebo response rates in IBD, both ulcerative colitis (UC) and Crohn´s Disease (CD), are somewhat lower and in the range of 15 to 30%, depending on whether the endpoints were clinical benefit (improvement) or remission (based on standardized, *e.g.* endoscopic or histological criteria) and whether the studies were to initiate or to maintain remission.

**Table 1:** Systematic Reviews and meta-analyses of placebo response rates in different functional and non-functional gastrointestinal diseases.

Author, Year	Ref No	Clinical Condition	Number of studies/patients	Pooled Placebo Response (%)
Pitz et al., 2005	(5)	IBS	53/6326	36 (global improvement)
Pitz et al., 2005	(5)	IBS	39/5445	28 (abdominal pain)
Patel et al., 2005	(6)	IBS	45/3352	40.2
Dorn 2010	(26)	IBS	19/658	42.6 (CAM treatment)
Ford et al., 2010	(14)	IBS	73/8364	37.5
Allescher et al., 2001	(19)	NUD	29/1373	37.2/46.6^###^
Ilnyckyj et al., 1997	(27)	IBD-UC*	16/11/8	26.7/30.3/25.2
Su et al., 2004	(28)	IBD-CD**	21/327	18
Jairath et al., 2017	(29)	IBD-UC^+^	57/4062	19/22–10/33
Jairath et al., 2017	(30)	IBD-CD^++^	100/7638	32/26–18/28
Estevinho et al., 2018	(31)	IBD (UC,CD)^#^	26/2842	17.7/27.5–13.2/27.6
Ma et al., 2018	(32)	IBD-UC^##^	64/5282	14/20–23/35
Macluso et al., 2018	(33)	IBD-UC	31/2702	9/34/26°
Duijvestein et al., 2019	(34)	IBD-CD	5/188	16.2/5.2°°
de Craen et al., 1999	(35)	DU	79/1350	44.2/36.2°°°
Cremonini et al., 2010	(36)	GERD	24/3041	18.9

IBS, irritable bowel syndrome; NUD, non-ulcer dyspepsia; IBD, Inflammatory Bowel Disease; UC, Ulcerative colitis; CD, Crohn’s Disease; CAM, Complementary and alternative medicine; DU, Duodenal Ulcers; GERD, Gastroesophageal reflux disease.

*data for % clinical benefit, not for % remission, with clinical, endoscopic, or histological endpoints, respectively. For % remission the respective values are 9.1, 13.5, and 8.6%.

**CDAI as endpoint, % received remission (CDAI score decrease varied from >50 to >100).

^+^data for maintenance trials (N = 9) and for induction trials (N = 48); endpoint remission and clinical response rates for maintenance and induction, respectively, are listed.

^++^data for maintenance trials (N = 40) and for induction trials (N = 67); endpoint remission and clinical response rates for maintenance and for induction, respectively, are listed.

^#^Quality of life improvement (IBDQ, SF36); data for IBDQ, separated for induction and maintenance in UC and CD, respectively.

^##^data for maintenance trials (N = 8) and for induction trials (N = 56); endpoint remission and clinical response rates for maintenance and induction, respectively, are listed.

^###^Response rates for prokinetics vs acid-suppressing treatments are given.

°induction rates for remission, response, and mucosal healing, respectively, are given; for maintenance, the respective data are 14, 23, and 19%.

°°placebo response for endoscopic improvement and remission, respectively.

°°°placebo response data for 4/day versus 2/day regiments are given.

However, it is evident from these data that in chronic, recurrent diseases as IBD the placebo response also includes cases of spontaneous remission of the disease and are not easily separated from these—for this, “no treatment control groups” would be needed and that definitively is not possible in severe and life-threatening diseases such as UC and CD, while it would be possible (but never has been done) in IBS. Since spontaneous waxing and waning of symptoms is also a characteristic on FGD, care has to be taken not to overinterpret the placebo response rates in IBS by ignoring spontaneous symptom variation and others, *e.g.* methodological contributions to the placebo effect in RCT. Across many mild or minor diseases, this has been done by some authors (37–39), and they estimated these contributions to explain 50% of the placebo effect.

Not surprisingly, some meta-analyses in IBD have used the placebo response rates in drug RCT to rather calculate the relative risk of disease recurrence and relapse in maintenance studies with IBD and found an increased risk compared to drug in the range of 23.7% in CD patients after surgery (40), while others (41) found the relapse rate in gastric ulcer studies to be 3.29% higher with placebo as compared to the (acid suppressing) drug. This is not to mix up the so-called nocebo effects (42, 43) with the reports of adverse events (AE) while on placebo during a double-blinded RCT, although this as well is difficult to separate without adequate control groups, *e.g.* register studies that include a “monitoring only” arm (12) (see below).

### B: Is the Placebo Effect in FGD Different From Other Functional Non-GI Disorders?

The question specifically addresses pain syndromes, as (visceral) pain is the central characteristic of most FGD, although it is admitted that among the very many functional syndromes the Rome Committee has identified—altogether 38 in the Rome III edition—some are not associated with pain but rather with disturbed bowel function (motility) only. However, pain is a prerequisite to receive the diagnosis of IBS, and only data on placebo effects and responses in IBS are in the focus and have been studied extensively.

The already mentioned systematic reviews and meta-analyses (25) listed quite a number of functional syndromes outside the gastrointestinal tract in which the placebo effect has been studied. **Table 2** summarized these data but restricted the studies to those meta-analyses of trials in painful clinical conditions. Three not pain-associated diseases (overactive bladder syndrome, OAB; premenstrual syndrome, PMS; chronic fatigue syndrome, CFS) are single examples listed for comparison.

**Table 2:** Placebo response rates in different clinical pain conditions.

Author, Year	Ref No	Clinical Condition	Number of studies/patients	Pooled Placebo Response: % or ES or SMD
Diener et al., 1999	(44)	migraine	15/1345	25.9 (44/13)*
Macedo et al., 2008	(45)	migraine	98/11793	9/18/28/32**
Macedo et al., 2006	(46)	migraine	32/1416	21
Ho et al., 2009	(47)	migraine	8/1322	36.2/38.1–9.5/10.5***
Meissner et al., 2013	(48)	migraine	79/2828	22/26/23/38/24^+^
Quessy et al., 2008	(49)	NP	35/3265	26.5–15.5^++^
Zhang et al., 2008	(50)	osteoarthritis	193/16364	ES: 0.51/0.77^+++^
Häuser et al., 2011	(51)	fibromyalgia/DNP	72	SDM: 0.42/0.72 (45/62)^#^
Capurso et al., 2012	(52)	pain/pancreatitis	7/202	19.9
Chen et al., 2017	(53)	osteoarthritis	124/15633	ES: 0.52
Athayde et al., 2018	(54)	pouchitis	12/229	47/24°°°
Huang et al., 2019	(55)	osteoarthritis	21	SMD: −0.16−0.34/−0.31°°
Porporatti et al., 2019	(56)	TMD	42/1657	29/19/26°
Freeman et al., 1999	(57)	PMS	2/247	33
Cho et al., 2005	(58)	CFS	29/985	19.6 (14/16.5/24)^###^
Lee et al., 2009	(59)	OAB	36/5735	PE: −1.15/−1.27/12.4^##^

NP, Neuropathic pain; DPN, diabetic polyneuropathy; TMD, temporomandibular disorders; PMS, premenstrual syndrome; CFS, chronic fatigue syndrome; OAB, overactive bladder; ES, effect size; SMD, standardized mean difference.

*overall, headache response and pain-free response are listed.

**responses for 30 min, 1, 2, and >2 h after intake.

***for pain relief and pain free in females/males.

^+^for oral pharmacologic, CAM, injection therapies, sham acupuncture/surgery and sham CBT/electromagnetic stimulation therapies, respectively.

^++^for NP in DPN and post-herpes neuropathy, respectively.

^+++^ES estimate: Clinically, an ES of 0.2 suggests a small effect, 0.5 means a moderate effect, and 0.8 and over indicates a large effect. ES are given for all trials and for three trials comparing treatment with a no-treatment control, respectively.

^#^SDM for DNP and fibromyalgia, respectively, are given; percentage relates to the improvement in the active group that can be attributed to placebo.

^##^Point estimated (PE) from meta-analysis for incontinence episodes, micturition frequency, and voiding volume, respectively, are given (all highly significant).

^###^Overall response and response in low, medium, and high expectation groups, respectively.

°response for laser therapy, drugs, and other therapies.

°°SMD for patient-reported outcome (PRO) for pain, muscle strength, and range of motion.

°°°for induction and maintenance trials, respectively.

As can be seen, painful non-GI clinical conditions are all associated with placebo response rates of 20% and higher, and up to 40% as is regularly the case in FGD, especially when visceral tissue is involved (pouchitis, pancreatitis); effect sizes are moderate to strong. A similarly strong placebo effect seems to dominate in pains associated with vascular mechanisms (migraine). Whether this moderate increase of visceral placebo analgesia over somatic placebo analgesia is a consequence of the rather diffuse nature of visceral pain, its specific characteristic as being deep, dark, and poorly locatable, or specifics of the “personality” of the patients affected cannot be answered from such meta-analyses.

### C: Is the Placebo Effect in FGD Related to Effects Seen in Psychiatry?

The area in which placebo effects and their determinants are best investigated is psychiatry, and it was psychiatry in which increased placebo response rates in RCT were first noted—in fact, drug development has occasionally been hindered by too strong placebo effects rather than by weak drugs (60, 61). It was psychiatry as well where the first evidence of increasing placebo effects over time were noted. It is, therefore not surprising that increased placebo response rates in non-psychiatric, *e.g.* gastrointestinal conditions were attributed to psychiatric comorbidity in otherwise somatically affected patients. This is specifically true for FGD of IBS-type, as the overall placebo response rate in IBS is 40% (as discussed above), and the overall response rates in depression trials match these 40% quite well (62).

A similar systematic review of meta-analyses than the one introduced above for all medical conditions (25) summarized the placebo effect in RCT in psychiatric disorders recently (63). Here it is discussed whether the various predictors of the placebo response especially in depression trials match findings from prediction analysis in IBS and FD. These predictors are classified into three groups: Disease characteristics, patient characteristics, and study design characteristics. To the best of our knowledge, only one regression analysis has been performed with data from the placebo arm of a trial with 599 patients with constipation-predominant IBS (64) to identify predictors of the placebo response, as well as predictors of a non-response to placebo in the same trial,

#### Disease Characteristics

The overwhelming finding from most such prediction analyses is that patients with a lower symptom severity at study start will show stronger placebo effects than patients with more severe symptoms (25). This may reflect the tendency of drug companies to recruit a mildly-to-moderately affected patient population for their studies, but it may as well reflect the fact that patients with milder symptoms may be more willing to participate in a placebo-controlled therapy trial. The downside of this tendency is that RCT may not represent the medical reality in terms of patients tested, and this may corroborate the representativeness of the study results. It has been noted that drugs such as serotoninergic antidepressants are by far less effective in daily routine than had been reported in RCT (65). However, this may as well be due to overinterpretation of the RCT data. A lower symptom severity is often associated with a shorter disease history that was found to predict higher placebo responses, while previously untreated patients were sometimes found to generate higher responses but in other cases, lower responses. Rather than previous treatment *per se*, treatment success or failure may determine the response to subsequent trials. Another of the concerns related to the representativeness of antidepressant trials is that patients recruited may have been taken off their regular medicines and may have experienced symptom worsening before being included into a RCT, and thereby the gap between drug and placebo arms may have been artificially widened.

The only meta-analysis that has studied the prediction in IBS (14) did not find disease severity to affect placebo responses, mainly because patient definition for recruitment was based on the IBS diagnostic criteria (Rome) that regularly do not include assessments of symptom severity, *e.g.* by the IBS-SSS score (66). The different Rome criteria used over time did not result in differences of the placebo effect (14). The re-analysis of the data from a single IBS-C RCT (64), confirmed that placebo responders had lower baseline pain severity than non-responders, and that a pain response as early as week two of the trial was associated with a higher placebo response with respect to the primary endpoints, a >30% pain relief and “adequate pain relief”; the latter response was also associated with a placebo response for spontaneous bowel movements. A higher number of baseline spontaneous bowel movements were associated with lower placebo response.

For functional dyspepsia, two re-analyses of individualized data from RCT can be used to answer this question: in one (67), a lower symptom burden at baseline and a symptom increase during run-in were associated with higher placebo responses, while in the other (68) this could not be confirmed; instead, an unstable symptom pattern was predictive of higher placebo responses as was a higher Body Mass Index (BMI). The BMI data are probably accidental findings in this specific cohort as was the smoking status in the other one (67), or it may represent a more general feature (69) yet to be explored.

#### Patient Characteristics

At least in some conditions, especially in psychiatric diseases, younger patient age was usually associated with higher placebo response rates (25) and has led to speculation about reasons for this (70) that are not conclusive overall; however, conflicting evidence exists as well. On the contrary, the widely believed idea that women may generate higher placebo response rates in RCTs was not supported by our analysis of clinical trials (25), while experimental placebo studies tend to confirm sex difference, albeit in both directions: Men seem more prone to show placebo responses in expectancy-based designs, while women responded stronger in learning (conditioning) experiments—the reasons for this difference are discussed elsewhere (71).

Age and sex were not reported to drive the placebo effect in the largest meta-analysis (14), while at least one (5) noted younger age to be associated with higher placebo effects in IBS. Data from FD studies (67, 68) did not find evidence for the influence of age and sex. Other patient characteristics, especially personality variables, are usually not assessed in RCT outside psychiatry because of the risk of limiting the indication of the drug under investigation. And pieces of evidence from experimental trials (72) have never been confirmed in clinical studies.

#### Study Design Characteristics

Probably the most consistent and surprising finding in psychiatric and neurological trials is the fact that the so-called “unbalanced randomization” determines the placebo effect: with a higher chance to receive active treatment in enrichment trials, trials with more than one drug arm, different dosages, comparator trials or trials which attempt to motivate more patients in general, the placebo effect rises [*e.g.* (44, 73), for a discussion see (63)]. It is of utmost importance to note that this feature has not been replicated in IBS studies at all (3). However, in IBS such studies were usually multi-center trials with large patient populations conducted by the pharmaceutical industry (see **Figure 2**), while in psychiatry, many such trials were small scale with an *a priori* risk of high placebo effects.

Another feature that has steered the placebo discussion, especially in depression, is an increase of the placebo effect over time, noted as early as 2002 (74). This is counter-intuitive towards the fact that more recent studies tend to be longer, and that shorter trial duration usually was found to be prone to higher placebo response rates (63). In depression, this trend was at least questioned (75–77), and it was not confirmed in IBS trials either (3) Neither was it found in IBS studies that trials between the US and Europe differed in the placebo effect (with higher responses in the US in depression), and with industry-initiated studies producing higher placebo effect than investigator-initiated studies, as was the case in some psychiatric trials (63).

One characteristic that was similar between psychiatric trials and FGD trials is the number of planned study visits during a trial: the more visits are planned the higher is the placebo response, a feature that was not only found in IBS (5, 6)—though with conflicting trends, see (78) —but also in IBD trials (27).

A very specific drug design feature in FGD (IBS), requested by the European Medical Agency (EMA) and matched by neither the Federal Drug Administration (FDA) rules in the US nor the EMA/FDA rules in any other class of diseases, is to either conduct long-term (*e.g.* six months) trials or to conduct a short-term (week) trial and repeat the treatment after re-randomization for another short-term to verify the drug is still effective. To the best of our knowledge, only one IBS RTC has been conducted with the second option (79) and showed the placebo effect in the second treatment period to be of similar size than in the first treatment phase. However, most of the assessment tools for outcome measures used have not been validated for such test strategy, but this also holds true for other endpoints, *e.g.* the “global assessment of improvement” (GAI) for 6-month trials in IBS.

In summary of this part of the review therefore, none of the study characteristics (disease, patient, and design) driving the placebo response in psychiatry seem to contribute to the placebo effects seen in FGD, and except for the number of study visits and the effects of symptom severity at baseline, seem to be of relevance in IBS. Hence, the prediction capability of placebo meta-analyses remains to be rather poor in FGD in gastroenterology.

### D: What Are the Consequences of These Findings for Future Drug Trials in FGD?

A number of immediate conclusions can be drawn from the above discussed data:

- For one, the placebo effect in RCT in FGD, especially in IBS, may be slightly higher than in other functional and organic diseases, but with around 40% it is not extraordinarily high as long as the sample size is sufficiently high (say: more than 100 patients per study arm). Studies with lower sample sizes should be avoided.
- Patient reported outcomes usually produce higher placebo response rates than biomarker readouts as is evident *e.g.* from differences between symptomatic readouts and endoscopic/histological endpoints. It would therefore be advisable to add one or more biomarkers to IBS studies, currently relying on symptom reports in diaries mainly.
- Because time trend observed in psychiatric and neurological disorders (increased placebo response rates in more recent RCT) has not been confirmed in FGD, this underlines the importance to maintain current patient definition and endpoint selection in IBS trials as manifested in the Rome criteria.
- Since unbalanced randomization appears not to be a factor influencing the size of the placebo response, such strategies, *e.g.* adding comparator drugs to a trial, should be encouraged in gastroenterology, especially in FGD where they are literarily non-existing. On the other hand, enrichment trials to enhance the drug effects and to limit placebo effects, as they become popular in psychiatry, seem not to be needed in FGD in gastroenterology.
- A trial length of 12 weeks seems to be reasonable, as longer trials do not eliminate the placebo effect, as was previously hoped, but shorter trials definitively carry the risk to increase placebo response rates.
- Unbalancing the sex ratio in RCT with IBS and FD patient may be a risk factor for sex-related drug effects, but is obviously not affecting the placebo effects. There is, however, evidence for different placebo responses in relation to age that should be kept in mind when planning a RCT.

Eliminating placebo-controlled studies and replacing them with comparator trials (also called head-to-head trials) do not eliminate the placebo effect but make it more difficult to identify and quantify it—placebo effects are immanent to all medical and psychological therapeutic interventions and may also affect diagnostic procedures (43, 80, 81). It has been shown (*e.g.* in psychiatry) that in fact the 100% chance to receive active treatment may drive the placebo effect to another height (62). On the other hand, as has been argued above, a substantial fraction of what appears to be the placebo effect in RCT is in fact a contribution of spontaneous symptom variation and—in chronic recurrent diseases—remission and relapse. To control both, the placebo effect without placebo provision and the contribution of the spontaneous course of the disease, in “real world studies” (studies under realistic conditions in daily medical routine), other measures may be needed that are discussed in more detail elsewhere (12, 19):

- From a methodological standpoint, even comparator trials comparing two or more drugs, the novel one and the one already on the market, should always include a placebo arm as well, to allow testing the non-inferiority of the novel compound against the established one as well as its superiority against placebo.
- To include a “no treatment” control arm into conventional placebo-controlled trials, studies should make use of the “cohort multiple randomized controlled trial” (CMRCT) (82), also called “Zelen design” (83): The “monitoring only” study is separated from the interventional study recruitment, *e.g.* by using large cohorts in disease registries. Patients recruited to participate in disease monitoring are subsequently asked to volunteer for the interventional part, and those not agreeing remain in the monitoring arm for control purposes.
- With open-label observational studies and no apparent randomization, monitoring spontaneous symptom variation can also be achieved by utilizing the same strategy, called “controlled open-label trial” (COLT) discussed in a recent paper (12).
- Finally, open label placebo treatment (84) can be added to a conventional placebo-controlled trial, either with full double-blinded randomization (85) or allowing patient preferences for either arm, what has been called preference design (19).

## Summary and Conclusion

Contrary to common belief and discussion, the placebo effect seen in RCT in FGD is not specifically high and extraordinary as compared to other comparable (*i.e.* functional) disorders. It shares less than expected commonalities with the placebo effect in psychiatry, and very few predictors have yet been identified that determine its effect size, especially very few that are driven by design features of the studies. Current practice of RCT in IBS seems to limit and control the placebo effect quite well, and future trial practice, *e.g.* head-to-head trial still offers options to maintain this control even in the absence of placebos used.

## Author Contributions

SK and PE conceptualized the paper. PE wrote the paper. SK corrected and approved the manuscript.

## Conflict of Interest

The authors declare that the research was conducted in the absence of any commercial or financial relationships that could be construed as a potential conflict of interest.
